# Global quantitative analysis of the human brain proteome in Alzheimer’s and Parkinson’s Disease

**DOI:** 10.1038/sdata.2018.36

**Published:** 2018-03-13

**Authors:** Lingyan Ping, Duc M. Duong, Luming Yin, Marla Gearing, James J. Lah, Allan I. Levey, Nicholas T. Seyfried

**Affiliations:** 1 Department of Biochemistry, Emory University School of Medicine, Atlanta, GA 30322, USA; 2 Center for Neurodegenerative Diseases, Emory University School of Medicine, Atlanta, GA 30322, USA; 3 Department of Pathology and Laboratory Medicine, Emory University School of Medicine, Atlanta, GA 30322, USA; 4 Department of Neurology, Emory University School of Medicine, Atlanta, GA 30322, USA

**Keywords:** Alzheimer's disease, Protein-protein interaction networks

## Abstract

Patients with Alzheimer’s disease (AD) and Parkinson’s disease (PD) often have overlap in clinical presentation and brain neuropathology suggesting that these two diseases share common underlying mechanisms. Currently, the molecular pathways linking AD and PD are incompletely understood. Utilizing Tandem Mass Tag (TMT) isobaric labeling and synchronous precursor selection-based MS3 (SPS-MS3) mass spectrometry, we performed an unbiased quantitative proteomic analysis of post-mortem human brain tissues (*n*=80) from four different groups defined as controls, AD, PD, and co-morbid AD/PD cases across two brain regions (frontal cortex and anterior cingulate gyrus). In total, we identified 11 840 protein groups representing 10 230 gene symbols, which map to ~65% of the protein coding genes in brain. The utility of including two reference standards in each TMT 10-plex assay to assess intra- and inter-batch variance is also described. Ultimately, this comprehensive human brain proteomic dataset serves as a valuable resource for various research endeavors including, but not limited to, the identification of disease-specific protein signatures and molecular pathways that are common in AD and PD.

## Background & Summary

Many neurodegenerative diseases are found to have common cellular and molecular mechanisms such as pathological protein aggregation and have been collectively termed as ‘proteinopathies’^1,2^. Alzheimer’s disease (AD) and Parkinson’s disease (PD) are two of the most common neurodegenerative diseases that share both clinical and pathological overlap. In the United States, AD affects approximately 10% people over 65, whereas PD affects 1–2% of all individuals over 60 (refs 3,4). AD is characterized by the accumulation of intracellular hyper-phosphorylated tau, neurofibrillary tangles and extracellular beta-amyloid (Aβ) plaques in the brain, which promote neuronal death, and ultimately dementia^5,6^. In contrast, PD is characterized as a chronic and progressive movement disorder with abnormally deposited Lewy bodies, comprised of aggregated α-synuclein (α-syn)^7^. Notably, Aβ, tau and α-syn aggregates can often co-exist and increase the incidence of clinical disease^8–13^. Following neuropathological examination, approximately 50% of AD cases harbor Lewy bodies^14,15^, and an equal percentage of PD cases have co-morbid AD pathologies (i.e., Aβ plaques and tau tangles)^16,17^. Despite the prevalence of shared pathology and clinical overlap, the molecular and cellular events linking AD and PD are incompletely understood. Here we report a large-scale quantitative proteomic dataset of AD and PD postmortem brain tissue that can be used to further our understanding of these related neurodegenerative diseases.

Advances in liquid chromatography coupled to tandem mass spectrometry (LC-MS/MS) now facilitate high-throughput detection and quantification of thousands of proteins in numerous clinical samples^18–20^. There are currently two prevalent strategies for protein based quantitation, referred to as either label-free or differential labeling based approaches. In general, label-free methods have been favored for quantitative proteomic studies of clinical samples due to their low cost and high-throughput^21,22^. To this end, we have recently employed a label-free approach to quantify ~3,000 proteins from over 100 human brain tissues to define signatures associated with neuropathological burden and cognitive dysfunction in AD^23^. However, label-free strategies have several limitations, including dependence on accurate mass measurements and reproducible peptide retention times by liquid chromatography (LC), which if not carefully controlled can introduce bias in peptide ion intensities^24–26^. These biases ultimately contribute to a higher number of missing values and increased variance of lower abundant proteins^24–26^. By contrast, metabolic labeling strategies such as stable isotope labeling with amino acids in cell culture (SILAC) and ^15^N-labeling are currently available and can alleviate these issues, but they have limited applicability because they require specialized growth conditions contributing to longer development time and increased cost^27^. More recently, the development multiplexed quantitation via isobaric chemical tags (e.g., tandem mass tags (TMT) and isobaric tags for relative and absolute quantitation-iTRAQ)^28^, circumvent the limitations of both metabolic labeling and label-free strategies^29^. Using a 10-plex TMT based approach, peptides are first derivatized with either one of ten different isotopically (^13^C- and ^15^N) labeled amine-reactive isobaric tags. Notably, these tags have identical chemical structure, yet have unique mass reporters ions that are used for MS/MS quantification^30^. This strategy enables the multiplexing of all peptide sets prepared from different clinical samples to be combined into a single LC-MS/MS analysis and thus improves throughput and the breadth of coverage by avoiding missing values that are common in label-free based quantification. Due to the isobaric nature of the tags, all shared peptides from the 10 samples exhibit the same biochemical properties (i.e., exact mass, and ionization efficiency and retention time). Only during MS/MS sequencing does each tag fragment and release unique reporter ions, which are then used for peptide quantitation. Despite their advantages, initial attempts to quantify isobaric tags at the MS/MS level have been hampered by co-isolation and co-fragmentation of interfering ions that results in inaccurate TMT ratios^31^. However, with the release of newer generation mass spectrometers such as the Orbitrap Fusion Tribrid and the development of the synchronous precursor selection (SPS)-based MS3 (SPS-MS3) method, the previous limitations of increased duty-cycle time and TMT reporter ion suppression effects are largely addressed^31,32^. Thus, when coupled with off-line peptide prefractionation, the SPS-MS3 quantification approach enables the accurate quantitation of thousands of proteins across many samples simultaneously for large-scale quantitative proteomic applications^33^.

In this dataset, we applied TMT labeling with SPS-MS3 to achieve comprehensive global quantitation of two brain regions (frontal cortex and anterior cingulate gyrus) from four different groups: healthy controls, AD, PD, and co-morbid AD/PD cases. In total, we identified 127 321 total unique peptides from over 1.5 million peptide spectral matches (PSMs), which mapped to 11 840 unique proteins groups. To our knowledge, this is one of the deepest human brain proteomes generated to date^34,35^. This comprehensive human brain proteomic dataset will serve as a valuable resource for understanding the molecular signatures and pathways that link pathologic mechanisms in both AD and PD.

## Methods

### Human brain tissue

Post-mortem tissues from the dorsolateral prefrontal frontal cortex (Frontal Cortex, Brodmann Area 9) and anterior cingulate gyrus (Cingulate, Brodmann Area 24) was obtained from the Emory Alzheimer’s Disease Research Center (ADRC) brain bank. Both regions are affected in AD, whereas Lewy body densities in anterior cingulate gyrus predict cognitive deficits in PD^36^. Postmortem neuropathological evaluation of amyloid plaque distribution was performed according to the Consortium to Establish a Registry for Alzheimer’s Disease (CERAD) criteria^10^, while the extent of neurofibrillary tangle pathology was assessed in accordance with the Braak staging system^37^. All AD cases met NIA-Reagan criteria for the diagnosis of AD (high likelihood)^38^. Diagnoses were made in accordance with established criteria and guidelines for PD^13^. Cases were classified as co-morbid AD and PD (AD/PD) when they met pathological criteria for amyloid plaque, neurofibrillary tangle and Lewy body burden. All case metadata including disease state, gender, race, apolipoprotein (ApoE) genotype, age of death, and post-mortem interval (PMI) are provided [Data citation 1].

### Brain tissue homogenization and protein digestion

Procedures for tissue homogenization were performed essentially as described^23^. In total, 40 samples across four pathological groups (*n*=10 per control, PD, AD and AD/PD groupings) were collected from each of the brain regions (frontal cortex and anterior cingulate); 31 of these cases had matched tissues across both brain regions [Data citation 1]. Approximately 100 mg (wet tissue weight) of brain tissue was homogenize in 8 M urea lysis buffer (8 M urea, 100 mM NaHPO4, pH 8.5) with HALT protease and phosphatase inhibitor cocktail (ThermoFisher) using a Bullet Blender (NextAdvance). Each Rino sample tube (NextAdvance) was supplemented with ~100 μL of stainless steel beads (0.9 to 2.0 mm blend, NextAdvance) and 500 μL of lysis buffer. Tissues were added immediately after excision and samples were then placed into the bullet blender (in 4 °C cold room). The samples were homogenized for 2 full 5 min cycles and the lysates were transferred to new Eppendorf Lobind tubes. Each sample was then sonicated for 3 cycles consisting of 5 s of active sonication at 30% amplitude followed by 15 s on ice. Samples were then centrifuged for 5 min at 15 000 g and the supernatant was transferred to a new tube. Protein concentration was determined by bicinchoninic acid (BCA) assay (Pierce). Prior to further processing, protein integrity and concentration accuracy was assessed by SDS-PAGE (Supplementary Figure 1). For protein digestion, 100 μg of each sample was aliquoted and volumes normalized with additional lysis buffer. Samples were reduced with 1 mM dithiothreitol (DTT) at room temperature for 30 min, followed by 5 mM iodoacetamide (IAA) alkylation in the dark for another 30 min. Samples were then 8-fold diluted with 50 mM triethylammonium bicarbonate (TEAB). Lysyl endopeptidase (Wako) at 1:100 (w/w) was added and digestion continued overnight. Trypsin (Promega) was then added at 1:50 (w/w) and digestion was carried out for another 12 h. The peptide solutions were acidified to a final concentration of 1% formic acid (FA) and 0.1% triflouroacetic acid (TFA) and desalted with a C18 Sep-Pak column (Waters). Each Sep-Pak column was activated with 1 mL of methanol, washed with 1 mL of 80% acetonitrile, and equilibrated with 2×1 mL 0.1% TFA. The samples were then loaded and each column was washed with 2×1 mL 0.1% TFA. Elution was performed with 2 rounds of 400 μL of 50% acetonitrile.

### Tandem mass tag (TMT) peptide labeling

Assuming complete digestion of all samples, an aliquot equivalent to 20 μg was taken from each sample and combined to make a global internal standard (GIS) per brain region. All peptides mixtures were dried down under vacuum. For each tissue region, 5 batches of 10-plex TMT kits (ThermoFisher) were used to label the 40 samples and 10 GIS mixtures. Sample arrangement is shown in Supplementary Tables 1 and 2. In each batch, TMT channels 126 and 131 were used to label GIS standards, while the 8 middle TMT channels were used to label 2 samples from each disease state. Labeling was performed according to manufacturer’s protocol. Briefly, each sample (80 μg of peptides each) was resuspended in 100 μL of 100 mM TEAB buffer. The TMT labeling reagents were equilibrated to room temperature and 41 μL anhydrous acetonitrile was added to each reagent channel and softly vortexed for 5 min. Peptide suspensions were transferred to the corresponding TMT channels and incubated for 1 h at room temperature. The reaction was quenched with 8 μl of 5% hydroxylamine. To ensure complete labeling select channels from each batch were analyzed by LC-MS/MS according to previously published methods^39^. All 10 channels were then combined and dried by vacuum to~500 μL. Sep-Pak desalting was performed and the elution was dried to completeness.

### Electrostatic repulsion-hydrophilic interaction chromatography (ERLIC) fractionation

Dried samples were resuspended in 100 μL of ERLIC buffer A (90% acetonitrile with 0.1% acetic acid) and loaded onto a PolyWAX LP column (20 cm by 3.2 mm packed with 300 Å 5 μm beads from PolyLC Inc) as reported previously^35^. An Agilent 1100 HPLC system consisting of a degasser, a binary pump, an autosampler, a microflow UV detector, and a fraction collector was used to carry out the fractionation. The gradient was from 0 to 50% ERLIC buffer B (30% ACN with 0.1% FA) over 45 min. A total of 44 fractions were collected and then combined to 21 fractions. Final fractions 1 to 20 consisted of alternating combinations (1 and 21, 2 and 22, etc.) and fraction 21 consisted of the last fractions (41 to 44) as previously described^35^.

### LC-MS/MS and TMT data acquisition on an orbitrap fusion mass spectrometer

Assuming equal distribution of peptide concentration across all 21 ERLIC fractions, 40 μL of loading buffer (0.1% TFA) was added to each of the fractions and 2 μL (2 μg equivalent) was separated on 25 cm long 75 μm ID fused silica columns (New Objective, Woburn, MA) packed in-house with 1.9 μm Reprosil-Pur C18-AQ resin (Dr Maisch). The LC-MS platforms consisted of a Dionex RSLCnano UPLC coupled to an Orbitrap Fusion mass spectrometer with a Flex nano-electrospray ion source (ThermoFisher). Sample elution was performed over a gradient of 3 to 30% Buffer B (0.1% formic acid in ACN) over 105 min, from 30 to 60% B over 20 min, and from 60 to 99% B over 5 min at 300 nl. The column was reconditioned with 99% B for 15 min at a flow rate of 500 nl/min and equilibrated with 1% B for 15 min at a flow rate of 350 nl/min. The Orbitrap Fusion (Thermo Scientific) was operated in positive ion data dependent mode with synchronous precursor selection (SPS)-MS3 analysis for reporter ion quantitation. The full scan was performed in the range of 380–1500 m/z at nominal resolution of 120 000 at 200 m/z and AGC set to 2×10^5^, followed by selection of the most intense ions above an intensity threshold of 5000 for collision-induced dissociation (CID)-MS2 fragmentation in the linear ion trap with 35% normalized collision energy. The isolation width for the frontal cortex samples was set to 1.5 m/z with a 0.5 m/z offset. For the anterior cingulate samples, the isolation width was set to 0.7 m/z with no offset. The top 10 fragment ions for each peptide MS2 was notched out with an isolation width of 2 m/z and co-fragmented to produce MS3 scans analyzed in the Orbitrap at a nominal resolution of 60 000 after higher-energy collision dissociation (HCD) fragmentation at a normalized collision energy of 65%. Of note, one sample (fraction 19) in Batch 1 from the cingulate gyrus was run in technical replicate. All resulting raw files (*n*=211) are provided [Data Citation 2].

### Protein identification and quantification

Raw data files from Orbitrap Fusion were processed using Proteome Discover (version 2.1). MS/MS spectra were searched against the UniProtKB Human proteome database (90 411 total sequences) [Data Citation 3] as previously reported^35^. We chose to include both Swiss-Prot and TrEMBL sequences in the database as the additional depth provided by ERLIC fractionation enables the sequencing of rare protein isoforms (i.e., proteoforms) that may not be appreciated in human brain. Despite significantly more protein entries, searching with the entire UniprotKB database only added ~5% more unique protein groups compared to searches against the Swiss-Prot database alone (42 179 target sequences; downloaded on 11/18/2017). The respective FASTA database used in this study is deposited in the on the Synapse (www.synapse.org). SEQUEST parameters were specified as: trypsin enzyme, two missed cleavages allowed, minimum peptide length of 6, TMT tags on lysine residues and peptide N-termini (+ 229.162932 Da) and carbamidomethylation of cysteine residues (+ 57.02146 Da) as fixed modifications, oxidation of methionine residues (+ 15.99492 Da) and deamidation of asparagine and glutamine (+0.984 Da) as a variable modification, precursor mass tolerance of 20 ppm, and a fragment mass tolerance of 0.6 daltons. Peptide spectral match (PSM) error rates were determined using the target-decoy strategy coupled to Percolator^40^ modeling of true and false matches. Reporter ions were quantified from MS3 scans using an integration tolerance of 20 ppm with the most confident centroid setting. An MS2 spectral assignment false discovery rate (FDR) of less than 1% was achieved by applying the target-decoy strategy. Following spectral assignment, peptides were assembled into proteins and were further filtered based on the combined probabilities of their constituent peptides to a final FDR of 1%. In cases of redundancy, shared peptides were assigned to the protein sequence with the most matching peptides, thus adhering to principles of parsimony. The search results and TMT quantification are included [Data Citation 4].

## Data Records

All files have been deposited on Synapse (www.synapse.org).These include sample metadata (Data citation 1) all mass spectrometry raw files (*n*=211) across both brain regions (Data Citation 2), the FASTA database (Data Citation 3), and TMT protein quantification results (Data Citation 4).

The mass spectrometry proteomics raw files and data analysis files have also been deposited to the *ProteomeXchange* Consortium (http://www.proteomexchange.org/) via the PRIDE partner repository with the dataset identifier (Data Citation 5).

## Technical Validation

### Deep coverage of the human brain proteome

**Figure 1:**
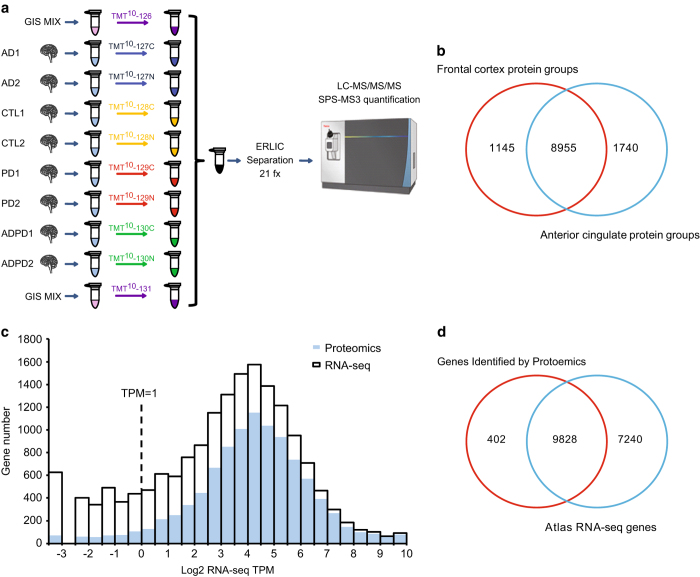
Deep coverage of human brain proteome in Alzheimer’s and Parkinson’s Disease. (**a**) TMT workflow. There were 40 tissue samples in both frontal cortex and anterior cingulate gyrus brain regions, which were TMT labeled across 5 individual batches per region. A total of 8 individual samples were labeled in every batch, 2 AD samples were dedicated to channels 127C and 127 N, 2 control samples were dedicated to channels 128C and 128 N, 2 PD samples were dedicated to channels 129C and 129 N, and 2 AD/PD samples were dedicated to channels 130C and 130 N. We also dedicated two TMT channels (126 and 131) to pooled global internal standards (GIS). After labeling, the samples were combined and fractionated by off-line ERLIC (*n*=21 total). Each fraction was analyzed and quantified by SPS-MS3 on an Orbitrap Fusion mass spectrometer. (**b**) There were 10 100 and 10 795 protein groups identified from frontal cortex and anterior cingulate gyrus, respectively, with 8,955 overlapping protein groups. Collectively, a total of 11 840 protein groups were identified across both brain regions. (**c**) Overlapping histogram comparing the degree of protein coverage for the highest and lowest expressed brain transcripts according to RNA-seq TPM bins. The open bar represents the distribution of protein coding gene numbers detected by RNA-seq, and the blue bar indicates the distribution of protein coding genes sequenced in this proteomic dataset. (**d**) Overlap between the proteome and RNA-seq data using identified gene symbols. This proteomic dataset covers ~58% of all detectable genes in the Human Brain Atlas RNA-seq dataset and 65% of highly expressed transcripts when considering RNA-seq TPM values above 1.

To reduce sample complexity and increase proteome depth, we employed off-line ERLIC fractionation prior to LC-MS/MS analysis (Fig. 1a). We used 10 batches (5 per brain region) of 10-plex TMT labeling kits, and separated these peptide mixtures into fractions for each batch followed by LC-MS/MS analysis on an Orbitrap Fusion mass spectrometer. Each batch had an equal number of control, AD, PD and AD/PD cases (2 per batch). Notably, two TMT channels in each batch were dedicated to global reference internal standards (GIS), representing an equivalent amount of pooled peptides from all cases analyzed in each brain region.

Following database search, a total of 127 321 unique peptides were identified that mapped to 11 840 protein groups at a 1% FDR on the peptide spectrum match (PSM) level across all samples, which represented 10 230 coding gene products (Fig. 1b). For each batch, ~8000 protein groups were identified. Total numbers of identified peptides, proteins and PSMs for both brain regions are listed in Tables 1 and 2.

To evaluate the depth of our data set, we compared the overlap of our proteomic data with available brain specific (cerebral cortex) RNA-seq data^41^ downloaded from the Human Protein Atlas website (Supplementary Table 3 and 4). RNA transcript abundances were calculated as transcripts per million (TPM) reads, which is measured by multiplying the estimated fraction of transcripts generated by a given gene. Notably, TPM is considered more comparable between samples of different origins and composition^42,43^. If we considered all genes identified by RNA-seq with no TPM cutoff, the 10 230 translated gene products identified in our proteomics analysis corresponds to ~58% (9828/17068) of all expressed genes found in the RNA-seq dataset (Fig. 1d). The proteins identified in the frontal cortex and anterior cingulate covered ~51% (8739/17068) and ~54% (9197/17068) of the expressed coding genes, respectively. To compare the degree of protein coverage for the highest and lowest expressed brain transcripts we generated an overlapping histogram. In general, the majority of abundant coding transcripts, within the range of 4–5 log_2_ TPM, were identified in our proteomic dataset. Notably, when enforcing a RNA-seq TPM filter cutoff of ≥1, which is typically employed to enrich for more reliably expressed genes^41^, the percent of translated gene products identified in the proteome increased to ~65% (9488/14541)^44^ (Fig. 1c). Surprisingly, we noticed that some highly expressed genes with very high TPM values were not identified. Upon further examination many of these gene lacked suitable tryptic peptides. For example, MT-ND4L has a log_2_ TPM value of 13.7, yet the translated protein (NADH-ubiquinone oxidoreductase chain 4L) only contains a single arginine available for trypsin cleavage resulting in 2 peptides that are not proteotypic^45^ (i.e., too long to be sequenced). Alternatively, there were also 402 gene-products identified in the proteome that were not identified by RNA-seq^23^ (Fig. 1d). Further analysis revealed that some of these proteins (n=114) had low coverage and were identified by only 1 PSM (Supplementary Figure 2) while others are related to blood^46^ and the lympathic system^47^. Gene Ontologies (GO) for all proteins that did not overlap with the transcriptome is provided in Supplementary Table 4.

**Figure 2:**
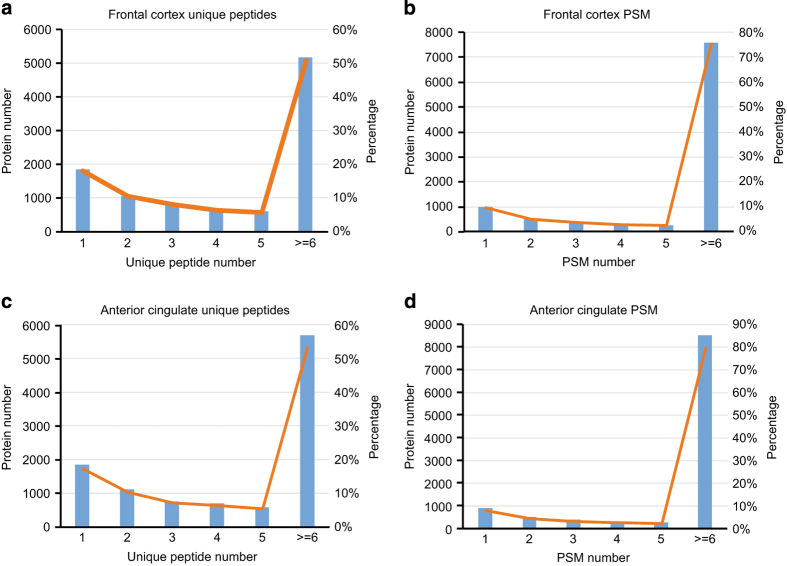
Assessment of peptide and peptide spectral matches for identified proteins across brain regions. (**a**) The frequency of unique peptides for all proteins identified from frontal cortex. More than 80% proteins identified with more than 2 unique peptides. (**b**) The frequency of peptide spectral matches (PSM) for all the proteins identified in frontal cortex. More than 75% proteins identified with more than 6 PSM, and about 85% proteins identified with more than 2 PSM. (**c**) The frequency of unique peptides for all proteins identified from the anterior cingulate gyrus. About 90% proteins identified with more than 2 unique peptides. (**d**) The frequency of PSMs for all the proteins identified in anterior cingulate gyrus. More than 85% proteins identified with more than 6 PSM. Both unique and shared peptide PSMs were considered in the analysis.

In data-dependent or ‘shotgun’ proteomics approaches, confidence of identification is directly related to the number of peptides and PSMs matched to a protein after database searching. In our dataset, more than 80% of proteins were identified by 2 or more unique peptides (Figs 2a and c) and more than 90% proteins were identified with at least 2 PSMs in both frontal cortex and cingulate gyrus (Figs 2b and d), which highlights the high identification confidence of this dataset. Collectively, these results show that prefractionation by ERLIC followed by LC-MS/MS on an Orbitrap Fusion can generate deep proteomes that cover the majority of expressed transcripts in human brain.

### Proteome wide quantitation utilizing two global reference internal standards

**Figure 3:**
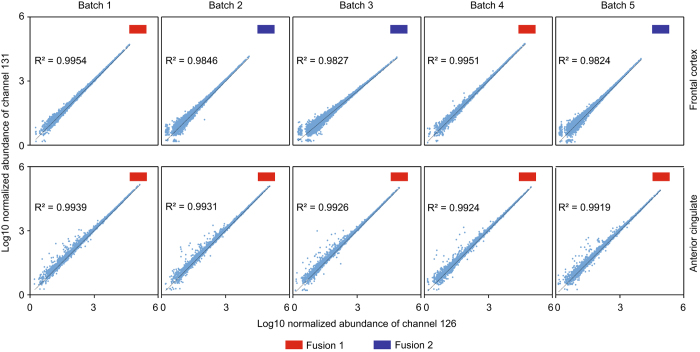
Correlation between brain region specific peptide reference standards across all batches. Two global internal standards (GIS) representing pooled peptides from all cases either from the frontal cortex or anterior cingulate gyrus were labeled with TMT channels 126 and 131. When comparing the normalized abundances from 126 and 131 reporters, the two channels showed strong linear correlation. The signals from Platform 1 (Orbitrap Fusion 1) had better correlation values than those from Platform 2 (Orbitrap Fusion 2).

**Figure 4:**
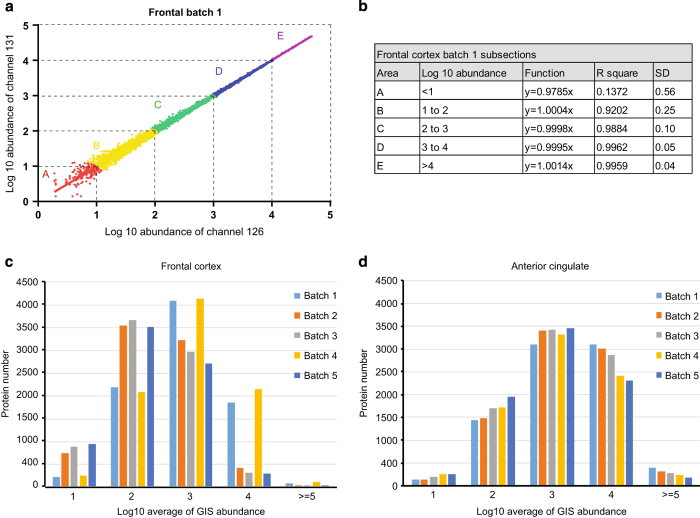
Proteome quantitation utilizing two peptide reference standards to asses intra- and inter-batch variability. (**a**) All the data was divided into 5 sections (A, B, C, D, E) according to the average normalized abundance from the GIS TMT channels (126 and 131). (**b**) Linear correlation for all five sections in Batch 1 from frontal cortex is represented. The correlation (R^2^) and standard deviation (SD) for each section was assessed using the log_10_ ratio of the GIS (TMT channel 126/131). The distribution of proteins according to normalized abundance of GIS in (**c**) frontal cortex and (d) anterior cingulate gyrus.

A major advantage of the TMT method is the ability to quantify proteins from multiple samples simultaneously. Each channel can be used to label different samples, which enabled the multiplexing of 10 individual samples in one LC-MS/MS analysis^28^. Typically, when analyzing more than 10 samples, a single TMT channel is dedicated as internal standard and included in each batch, which can be later used to normalize protein measurements within and across all samples^48^. Similarly, in this study, we pooled equivalent amounts of peptides from all individual cases (specific to brain region) to generate a GIS. However, we decided to dedicate two TMT channels (126 and 131) to the GIS (Fig. 1a) and required that both GIS TMT channels be readily quantified for protein quantification across the batch. This filtering criterion decreased the number of protein groups from 11 840 to 11 611 across both brain regions, the latter of which we considered quantifiable protein groups. The lack of consistent GIS measurements within the batch could indicate low signal abundance or the ubiquitous nature of product ion selection^49^. The GIS also allowed us to assess the intra-batch variability as these channels should show the same exact abundance after technical normalization. Indeed, TMT channel 126 and 131 reporter ion signals for identified proteins were very consistent and displayed an excellent linear relationship across batches (Fig. 3). However, there are some proteins that exhibited large variation in measurements, especially those with low abundance (Fig. 3). To enhance quantitative accuracy, we decided to implement filtering criteria based on the population wide standard deviation (SD) of the two GIS measurements. Based on the central limit theorem, the log_2_ ratio for the two GIS channels (log_2_ TMT channels 126/131) for all proteins measured should fit a standard Gaussian distribution with the mean at or near zero and SD representing the technical variation in protein measurement^50,51^. Thus, the SD allows one to evaluate the accuracy of all protein measurements within each batch. Since the SD differed based on protein intensity, we subdivided all GIS normalized abundances into 5 sections (per order of magnitude). For example, when assessing Batch 1 from the frontal cortex (Fig. 4a), proteins with the highest normalized abundance (>log_10_ of 1) displayed high correlation (R^2^ close to 1) and the smallest SD. However, proteins with lower normalized abundance of less than 10 (<log_10_ of 1) had higher variance in measurement (R^2^=0.1372) and a larger SD. Consistent with these observations, the SD across the 5 sections decreased with increasing normalized abundance (Fig. 4b). Correlation and SD values for each section from all 10 batches are listed in Supplementary Table 5. Since each protein in this dataset has its own GIS measurement, we considered them outliers if their GIS measurement was 4 SD away from mean value in any given subsection. However, this is a user defined criterion and can be differ based on the biological or research question at hand. Following filtering based on our criteria, the resulting protein number in all 10 batches are shown in Supplementary Table 6.

To assess quantitative consistency across batches, the distribution of proteins according to the average of two normalized GIS signal abundances was also investigated. For the anterior cingulate, the distribution was very similar across all 5 batches, with more than half of proteins generating signal abundances >100 (log_10_ of 2). However, in the frontal cortex dataset, the protein distribution between batches differed. Protein abundances in batch 1 and batch 4 showed a similar distribution to all batches derived from the cingulate cortex. In contrast, batches 2, 3 and 5, had a larger proportion of proteins with a reduced normalized abundance (Fig. 4c); more than 90% of proteins had higher average normalized GIS abundance in batches 1 and 4 compared to batches 2, 3, and 5. Since we prepared all the samples together, used all the same buffers and loaded same amount of peptides, we presume that the differences may come from platform variations (e.g. LC and/or mass spectrometer). The Emory Integrated Proteomics Core has two Orbitrap Fusion mass spectrometers that were used to analyze different batches of the frontal cortex dataset. Both instruments were equipped with the same exact Dionex LC and nano-column setup and had identical LC and MS parameter settings. Batches 1 and 4 were run on Platform 1 (Orbitrap Fusion1), whereas the other 3 batches were run on Platform 2 (Orbitrap Fusion2). Although the source of reduced signal intensities in Platform 2 is unclear, this difference informed our decision to analyze all TMT batches generated from the anterior cingulate on Platform 1, which resulted in higher signals and more identified proteins (Fig. 4d and Tables 1 and 2). Thus, by including the 2 GIS reference standards in each batch we could not only assess intra-batch variance in protein measurement, but also inter-batch variance due to platform differences. Furthermore, the two reference standards essentially serve as a null-experiment which allows investigators to filter outlier proteins measurements that don’t meet criteria for accuracy and precision as described above.

## Usage Notes

Ultimately, this comprehensive human brain proteomic dataset serves as a valuable resource for variety research endeavors including, but not limited to, the following applications:

### Use case 1: Protein expression

This dataset provides a quick and accurate reference for protein expression values, especially if an investigator wants to determine whether their protein of interest is abundantly expressed in human brain (Data Citation 4).

### Use case 2: Disease specific differential protein expression

The inclusion of control as well as AD, PD and AD/PD groups allows for the differential comparison of proteins across disease outgroups. Investigators can focus on comparisons that are specific to either AD or PD or those that are altered in both neurodegenerative diseases. These types of analyses could serve investigators working with cellular and/or other model organisms at AD and PD, who would be interested in whether a protein or pathway exhibits expression preservation in relevant human brain tissue (Data Citation 4).

### Use case 3: Protein Co-expression network analysis

More recently development in systems analysis approaches combined with deeper more comprehensive datasets such as this one, have enabled researchers the ability to look beyond standard differential expression analyses. Programs like WeiGhted Co-expression Network Analysis (WGCNA) and related alogrithms^52^, give investigators a system wide view of protein expression patterns that highlight groups of proteins (i.e., modules) which correlate to molecular and biological functionalities as well as cellular localization and cellular origin^23^. These programs can also readily correlate protein modules to case traits (clinical status and pathological burden) providing yet another level of analysis that can shed light on to the connection between protein co-expression and clinical diagnosis.

### Use Case 4: Identification and quantification of post-translational modifications (PTMs)

The phosphorylation of tau and α-syn are common pathological features in both AD and PD and are thought to have important roles in disease progression and pathogenesis^53,54^. Other PTMs have also been described for these proteins^55–57^. Thus, the deep coverage and quantitative accuracy in this dataset may make it possible to detect highly abundant disease-specific PTMs on key substrates in both AD and PD. Although enrichment strategies would provide a more complete PTM profile^51,58^, each type of modification requires a separate enrichment strategy that can be time consuming. This dataset provides a good starting point to identify the types of modifications that are most abundant for prioritization in future biological assays.

### Use case 5: Identification of novel brain-specific splice variants and coding changes

The raw data in this study could be used to search for alternative transcripts (e.g., splice junction peptides), alternative start sites and even single nucleotide variants (SNVs)^35^.These may show up in RNA-seq analysis of human brain and could be confirmed to be expressed at the protein level utilizing this current dataset.

### Use case 6: Selected reaction monitoring (SRM) or parallel reaction monitoring (PRM)

We have also supplied the peptide information (Data Citation 2), which includes more than 127 321 unique peptides, which can serve as a resource for PRM and SRM peptide candidate selection. This will complement currently available repositories such as the SRMAtlas^59^.

## Additional information

**How to cite this article:** Ping, L. Global quantitative analysis of the human brain proteome in Alzheimer’s and Parkinson’s Disease. *Sci. Data* 5:180036 doi: 10.1038/sdata.2018.36 (2018).

**Publisher’s note:** Springer Nature remains neutral with regard to jurisdictional claims in published maps and institutional affiliations.

## Supplementary Material



Supplementary Information

Supplementary Table 3

## Figures and Tables

**Table 1:** Protein identification from frontal cortex.

**Batch Number**	**Total Protein Groups**	**Total Unique Peptides**	**Total PSM**
1	7,647	60 347	123 567
2	7,471	56 508	120 175
3	7,353	53 402	114 437
4	8,010	67 981	139 217
5	6,964	50 345	115 336
Frontal cortex total	10 100	95 796	612 732

**Table 2:** Protein identification from anterior cingulate gyrus.

**Batch Number**	**Total Protein Groups**	**Total Unique Peptides**	**Total PSM**
1	8,234	78 021	198 586
2	8,390	78 378	183 496
3	8,549	80 072	186 347
4	8,079	72 849	184 850
5	8,262	74 593	180 673
Anterior Cingulate total	10 695	113 616	933 952
