# Supplementary material for: Global quantitative analysis of the human brain proteome in Alzheimer’s and Parkinson’s Disease
Source: Sci Data. 2018 Mar 13;5:180036. doi: 10.1038/sdata.2018.36 (PMC5848788; doi:10.1038/sdata.2018.36)
Supplement: Supplementary Information [file sdata201836-s2.pdf]

Supplementary Table of Contents

1) Supplementary Figure 1: SDS-PAGE analysis of brain homogenates. Page 1

2) Supplementary Figure 2: Protein-specific expression in human brain. Page 2

3) Supplementary Table 1: Sample arrangement for frontal cortex. Page 3

4) Supplementary Table 2: Sample arrangement for anterior cingulate gyrus. Page 4

5) Supplementary Table 3: Human Brain Gene Expression by RNA-seq.

6) Supplementary Table 4: GO analysis for 402 genes identified by proteomics but not by Human Atlas brain RNA-seq. Page 5

1) Supplementary Table 5: SD values in 5 subsections in all 10 batches. Page 6

2) Supplementary Table 6: Protein filter criterion. Page 7

Supplementary figure 1

a Frontal cortex

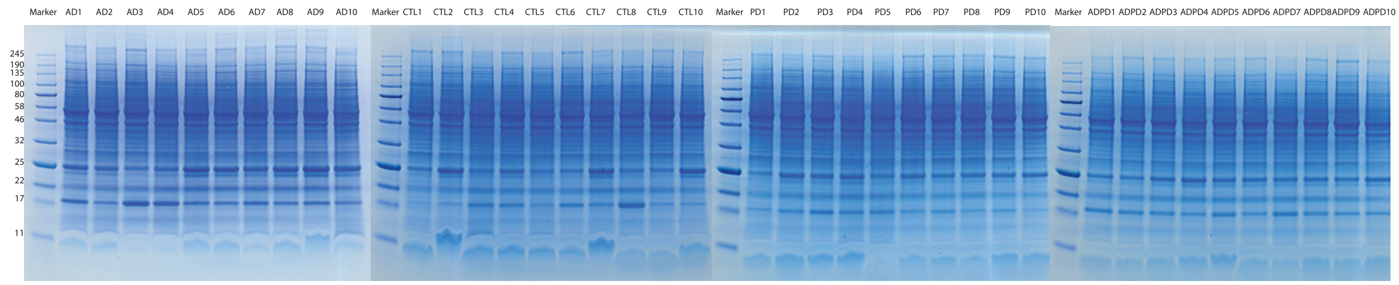

b Anterior cingulate

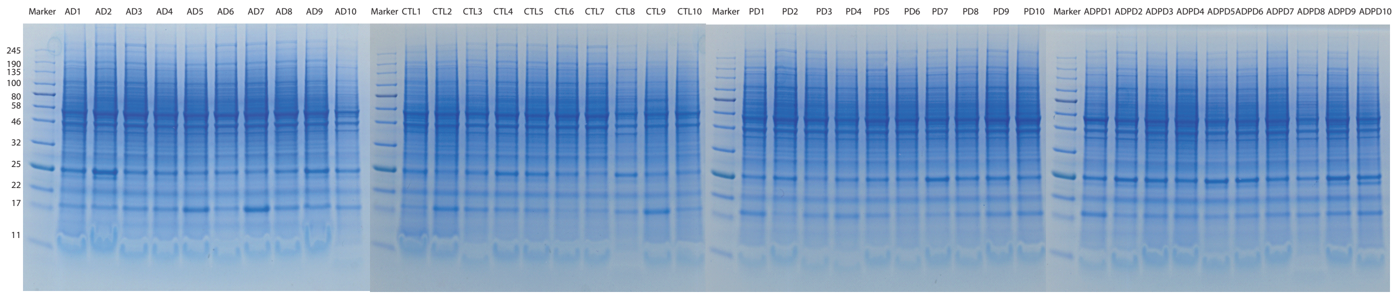

Supplementary figure 2

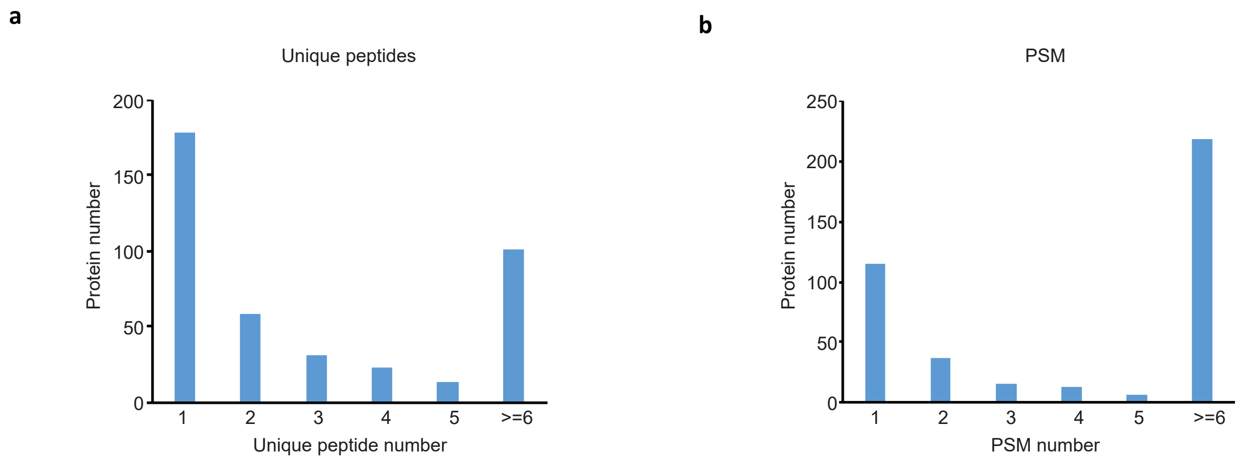

Batch Number	Machine	AD		Control		PD		ADPD	
		127C	127N	128C	128N	129C	129N	130C	130N
1	Fusion 1	E08-53	OS00-12	E05-130	E06-41	OS01-70	OS97-53	E06-128	E06-37
2	Fusion 2	OS00-32	OS03-163	A86-46	OS03-299	OS00-18	OS97-10	OS99-30	E05-179
3	Fusion 2	E04-186	E05-04	A93-03	OS02-35	E04-152	OS99-26	E05-132	OS00-28
4	Fusion 1	E06-155	E05-87	E08-101	A87-50	OS02-207	OS03-396	E04-68	E06-15
5	Fusion 2	OS00-11	OS98-11	OS00-06	E08-145	OS02-66	OS02-208	E06-13	E04-49

Supplementary table 1. Sample arrangement for frontal cortex.

Batch Number	Machine	AD		Control		PD		ADPD	
		127C	127N	128C	128N	129C	129N	130C	130N
1	Fusion 1	OS98-11	OS00-11	A86-46	A93-03	OS97-53	OS02-208	OS99-30	OS00-28
2	Fusion 1	OS00-32	OS03-163	OS00-06	OS02-35	OS03-392	OS03-395	E04-49	E04-68
3	Fusion 1	E04-186	E05-04	OS03-299	E05-74	OS03-396	E04-152	E05-132	E05-179
4	Fusion 1	E05-56	E05-87	E05-130	E06-41	E04-169	E05-81	E06-15	E06-37
5	Fusion 1	E06-155	E08-53	E08-101	E08-145	E09-136	E15-132	E06-128	E13-11

Supplementary table 2. Sample arrangement for anterior cingulate gyrus.

GO aspect	Z score	Ontology Name
biological_process	13.04	detection of chemical stimulus involved in sensory perception of smell
biological_process	11.7	humoral immune response
biological_process	11.25	neuropeptide signaling pathway
biological_process	10.48	protein activation cascade
biological_process	8.23	retina homeostasis
cellular_component	14.06	blood microparticle
cellular_component	9.47	keratin filament
cellular_component	8.97	extracellular region
cellular_component	7	extracellular space
cellular_component	6.13	membrane attack complex
molecular_function	13.04	olfactory receptor activity
molecular_function	11.1	phosphatidate phosphatase activity
molecular_function	10.91	G-protein coupled receptor activity
molecular_function	9.77	IgA binding
molecular_function	8.09	antigen binding

Supplementary table 4. GO analysis for 402 genes identified by proteomics but not by Human Atlas brain RNA-seq.

Brain Region	Batch Number	Log $_{10}$ normalized abundance				
		**<1**	**1 to 2**	**2 to 3**	**3 to 4**	**>4**
Frontal cortex	1	0.558	0.252	0.101	0.050	0.039
	2	0.658	0.271	0.094	0.045	NA
	3	0.654	0.269	0.097	0.042	NA
	4	0.615	0.237	0.102	0.051	0.041
	5	0.641	0.261	0.093	0.043	NA
Anterior cingulate	1	0.526	0.321	0.208	0.067	0.039
	2	0.644	0.336	0.194	0.070	0.039
	3	0.598	0.335	0.200	0.055	0.035
	4	0.600	0.346	0.185	0.066	0.038
	5	0.652	0.326	0.182	0.067	0.037

Supplementary table 5. SD values in 5 subsections in all 10 batches.

When the subsection protein count is less than 5, all proteins for that subsection is kept and no filter is applied. These subsections are given an “NA”.

Brain region	Batch NO.	1SD	2SD	3SD	4SD	No filter
Frontal cortex	1	6,075	7,938	8,268	8,335	8,357
	2	5,736	7,471	7,802	7,863	7,875
	3	5,640	7,352	7,719	7,769	7,780
	4	6,430	8,262	8,580	8,635	8,654
	5	5,374	7,040	7,348	7,406	7,420
Anterior cingulate	1	6,715	7,905	8,061	8,109	8,165
	2	6,867	8,074	8,221	8,266	8,316
	3	7,010	8,198	8,365	8,401	8,447
	4	6,625	7,695	7,861	7,900	7,935
	5	6,858	7,895	8,055	8,090	8,136

Supplementary table 6. Protein filter criterion

All the data in each batch was divided into 5 sections, which was filter by individual SD criterion. All the proteins were combined in each batch at different filter condition.
